# Targeting Glycolysis for Treatment of Breast Cancer Resistance: Current Progress and Future Prospects

**DOI:** 10.7150/ijbs.109803

**Published:** 2025-03-24

**Authors:** Zixu Niu, Jing He, Siyuan Wang, Bingjian Xue, Hao Zhang, Ruohan Hou, Zimeng Xu, Jing Sun, Fucheng He, Xinhong Pei

**Affiliations:** 1 Department of Breast Surgery, The First Affiliated Hospital of Zhengzhou University, Zhengzhou, 450052, China.; 2 Pharmaceutical College, Guangxi Medical University, Nanning, 530021, China.; 3 The First Clinical Medical College of Zhengzhou University, Zhengzhou, 450052, China.; 4 Department of Medical Laboratory, The First Affiliated Hospital of Zhengzhou University, Zhengzhou, 450052, China.

**Keywords:** Breast cancer, Tumor metabolism, Glycolysis, Targeted therapy, Drug resistance, Glycolytic inhibitors

## Abstract

Breast cancer stands as one of the most prevalent malignant tumors threatening women's health and is a leading cause of cancer-related mortality. Its treatment faces significant challenges, including drug tolerance and disease recurrence. Glycolysis serves not only as a critical metabolic pathway for energy acquisition in breast cancer cells but also essentially promotes tumor proliferation, invasion, metastasis, and the development of resistance to therapy. Recent studies have revealed a close association between glycolytic reprogramming and drug resistance in breast cancer, with high-level glycolysis emerging as a hallmark of malignancy, deeply involved in the initiation and progression of tumors. This review summarizes recent advances in research on key enzymes and signaling pathways regulating glycolysis within the bodies of breast cancer patients. It explores in depth these molecular mechanisms and their complex interaction networks, offering a fresh perspective on overcoming drug resistance in breast cancer. Moreover, it underscores the importance of developing specific inhibitors targeting key enzymes and regulators of glycolysis and suggests that combining such inhibitors with existing anticancer drugs could substantially enhance therapeutic outcomes for breast cancer patients and reduce the occurrence of drug resistance.

## Introduction

Breast cancer is the leading cause of cancer-related mortality in women, responsible for over 670,000 deaths globally in 2022, approximately 6.9% of all cancer deaths^1^,^2^. Its high incidence and mortality rates pose a significant threat to women's health^3^. Breast cancer is now understood to be a complex and heterogeneous disease^4^, categorized into various subtypes based on the expression levels of human epidermal growth factor receptor 2 (HER-2), progesterone receptor (PR), and estrogen receptor (ER)^5^. For patients expressing ER, PR, and HER-2, primary treatments include targeted medications that block HER-2 and endocrine therapies that interfere with hormone production^6^. Despite the efficacy of endocrine therapy and targeted therapy, overcoming treatment resistance and recurrence remains a critical research challenge. In some cases, patients continue to exhibit drug resistance and experience disease recurrence.

Glycolysis is a crucial metabolic process that helps tumor cells meet their energy and metabolic demands^7^. While mitochondrial oxidative phosphorylation is the primary energy source for normal cells in an oxygen-rich environment, tumor cells often exhibit a preference for glycolysis, even in the presence of oxygen, a phenomenon known as the "Warburg effect"^8^,^9^. This metabolic shift not only generates significant energy for tumor cell growth, survival, and proliferation but also provides the necessary metabolic intermediates for macromolecule synthesis^10^. Studies have shown a strong correlation between the reprogramming of glycolysis in breast cancer cells and the development of drug resistance^11^. Increased glycolysis levels are characteristic of malignant tumors, and this metabolic pathway plays a crucial role in tumor initiation and progression^12^. Therefore, understanding the role of glycolysis in breast cancer could lead to novel strategies to overcome drug resistance. This article provides a comprehensive review of recent research on the genes and signaling pathways involved in the regulation of glycolysis in endocrine therapy and targeted therapy-resistant breast cancer. By examining the mechanisms of action of these key molecules and their intricate interactions, we aim to offer novel perspectives on how to address drug resistance in breast cancer.

## Glycolysis in breast cancer

A growing body of evidence underscores the critical roles of glycolysis-related enzymes and pathways in breast cancer development and progression. For example, phosphoglycerate kinase 1 (PGK1) has been identified as a potential invasion promoter and survival marker through its regulation of the HIF-1α-mediated epithelial-mesenchymal transition (EMT) pathway^13^. Furthermore, a correlation exists between glycolytic efficiency and changes in phosphofructokinase-1 (PFK-1) in breast cancer cells^14^. PFK-1, a crucial rate-limiting enzyme in glycolysis, catalyzes the irreversible phosphorylation of F6P to F1,6BP. The enzymatic activity and expression of PFK-1 are tightly regulated through multiple molecular mechanisms. Notably, ubiquitin-specific protease 5 (USP5), a deubiquitinating enzyme, has been shown to stabilize the PFK-1 platelet isoform (PFKP) and upregulate its expression in breast cancer cells, thereby promoting glycolytic flux and supporting tumor metabolism. Similarly, pyruvate kinase 2 (PKM2) in breast cancer enhances glucose metabolism via the Let-7a-5p/Stat3/hnRNP-A1 feedback loop^15^. In addition to these enzymes, the expression of specific genes also plays a crucial role in modulating glycolytic activity in breast cancer. Current evidence suggests that long non-coding RNA (lncRNA) YIYA accelerates the conversion of glucose 6-phosphate, thereby stimulating glycolysis, cell proliferation, and tumorigenesis^16^. LncRNA DIO3OS promotes the glycolytic process in breast cancer by regulating the expression of lactate dehydrogenase A (LDHA)^17^. The metabolic characteristics of breast cancer can vary depending on the metastatic site. Brain and lung metastases often exhibit a glycolytic phenotype, while liver and bone metastases tend to have a non-glycolytic phenotype^18^. Beyond specific genes and their associated enzymes, other factors influence the glycolytic pathway in breast cancer. For instance, The Skp2-SCF E3 ubiquitin ligase complex plays a crucial role in regulating the PI3K/Akt signaling pathway by facilitating ubiquitination-mediated degradation of Akt. This regulatory mechanism has significant therapeutic implications, as inhibition of the PI3K/Akt pathway enhances tumor sensitivity to Herceptin^19^. The USF1-ATRAP-PBX3 axis activates the AKT/mTOR signaling pathway, promoting glycolysis and a malignant phenotype^20^. ATRAP interacts with ubiquitin-specific protease 14 (USP14) to deubiquitinate and stabilize the PBX3 protein in addition to augmenting aerobic glycolysis in breast cancer cells by upregulating the expression of glycolysis-related enzymes. Additionally, tumor-associated macrophages (TAMs) can enhance aerobic glycolysis and cell apoptosis resistance by delivering HIF-1α-stabilizing long non-coding RNA (HISLA) via extracellular vesicles (EVs)^21^.

Glycolysis-targeting therapeutic approaches offer a promising strategy to inhibit tumor growth and metastasis. In the glycolytic process, glucose transporters (GLUT1, GLUT3) facilitate glucose uptake into tumor cells, where it is subsequently metabolized into pyruvate by a series of enzymatic reactions^22^. Hexokinase (HK), phosphofructokinase (PFK), and pyruvate kinase M1/2 (PKM1/2) are key enzymes that regulate the rate-limiting steps of glycolysis. Pyruvate, the end product of glycolysis, is then reduced to lactate by lactate dehydrogenase (LDH) upon accepting hydrogen atoms from reduced nicotinamide adenine dinucleotide (NADH)^23^. The elevated levels of lactate produced by tumor cells serve as a precursor for biosynthetic processes and an energy source, fueling rapid cell proliferation and creating a tumor-promoting microenvironment^24^. For instance, lactate can modulate the expression of hypoxia-inducible genes, promote the accumulation of HIF-1α protein. HIF-1α, a pivotal regulator of aerobic glycolysis in tumor cells, not only supports tumor cell energy metabolism but also drives tumor angiogenesis by modulating the expression of angiogenic proteins, including VEGF.^25^. Oncogene activation or mutation, particularly in genes like *Ras*, *Src*, and *MYC*, can significantly upregulate the expression and activity of metabolic enzymes and transporters involved in the glycolytic pathway. For example, Shim et al. demonstrated that *c-Myc*, a highly expressed proto-oncogene, transcriptionally regulates *LDHA* expression in cancer cells^26^. The development and progression of cancer often involve the activation of various transcription factors, such as hypoxia-inducible factor-1 (HIF-1), tumor suppressor p53, and key signaling pathways, including PI3K/AKT/mTOR, RAS/RAF/MEK/ERK, and Wnt/β-catenin^27^-^29^. These factors can either directly or indirectly upregulate the expression of glycolysis-related genes, thereby enhancing glycolytic metabolism. For instance, Rankin et al. found that activated HIF-1 can control the expression of PGK1 and other glycolytic factors^30^. In addition, the PI3K/Akt/mTOR pathway induces the expression of GLUTs and glycolytic enzymes^31^, whereas p53 regulates glycolysis and GLUTs through mTOR and AMP-activated protein kinase (AMPK)^32^. Furthermore, the Wnt/β-catenin signaling pathway can regulate the activity of key glycolytic enzymes. Upon nuclear translocation, β-catenin binds to TCF/LEF transcription factors to directly activate the expression of genes encoding glycolytic enzymes^33^.

Angiogenesis and lymphangiogenesis abnormalities are significant indicators of tumor spread. Glycolysis and tumor angiogenesis and lymphangiogenesis are closely related in breast cancer. Through metabolic reprogramming, breast cancer cells enhance their glycolytic activity, resulting in substantial lactate production. This lactate is subsequently secreted into the tumor microenvironment via monocarboxylate transporter protein 4 (MCT4). Once absorbed by adjacent endothelial cells, lactate is converted into pyruvate, triggering the HIF-1α/NF-κB/IL-8 autocrine signaling pathway^34^. This activation promotes endothelial cell migration and angiogenesis. Concurrently, the high glycolytic activity within tumor cells creates a low-glycemic environment, leading to the upregulation of vascular endothelial growth factor (VEGF) expression in tumor tissue. Additionally, under hypoxic conditions, tumor cells secrete pro-angiogenic factors such as VEGF, which further amplify the expression of key glycolysis-related enzymes in endothelial cells^43^. This process initiates the glycolytic pathway, facilitating the formation of tip cells and lumen generation in nascent blood vessels. Tumor-associated fibroblasts (CAFs) in breast cancer contribute to angiogenesis through multiple mechanisms, including the release of stromal cell-derived factor-1 (SDF-1), recruitment of endothelial progenitor cells to tumor tissues, and stimulation of VEGF production by tumor cells^49^. Additionally, CAFs play a pivotal role in promoting breast cancer progression by facilitating the formation of new lymphatic capillaries from preexisting ones, a process known as lymphangiogenesis. While the direct effects of lactate on lymphangiogenesis remain poorly characterized, it indirectly influences this process by remodeling the tumor microenvironment and activating CAFs, thereby contributing to tumor dissemination.

**Figure 1:**
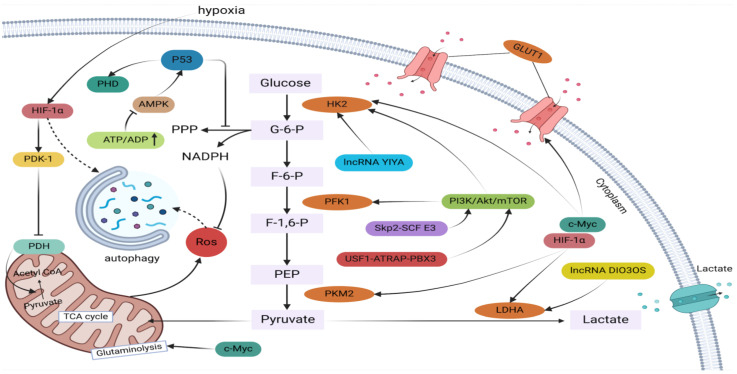
A complicated regulatory network regulates glycolysis in breast cancer. Lactate is produced by a sequence of enzyme processes from glucose. Increased NADPH generation , decreases ROS levels, which in turn reduces autophagy. The transcription factors c-MYC, HIF-1α, p53, and others control the expression of glycolytic genes in breast cancer cells in response to external stimuli such as tumor hypoxia, hypoglycemia, nutritional deprivation, and stress.

**Figure 2:**
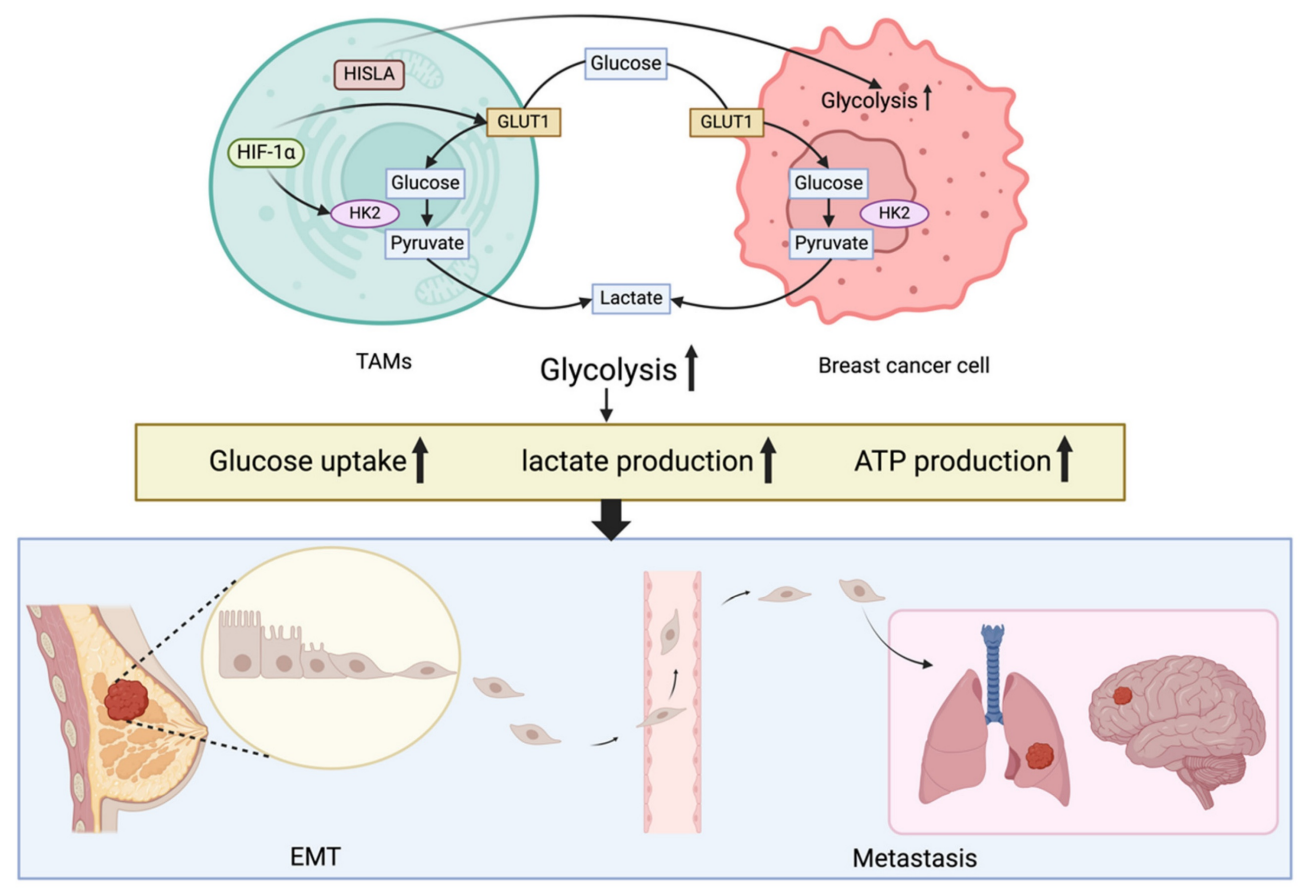
The intricate network of EMT and glycolysis in breast cancer cells. Activation of HIF-1α promotes glycolytic changes and increases glucose absorption and lactate release in breast cancer cells. Breast cancer cells exhibit enhanced aerobic glycolysis and anti-apoptosis when tumor-associated macrophages are presents, such as enhanced glucose uptake, lactate production and ATP production. Breast cancer cells that have spread by EMT to the brain and lungs are shown in the blue box; these metastases exhibit a glycolytic pattern.

In breast cancer resistance, glucose metabolism, specifically glycolysis, plays an indispensable role in maintaining cellular energetics. Therefore, targeting glycolytic pathways may have a substantial impact on the landscape of drug resistance observed in breast cancer therapy. Glycolytic transporters, enzymes, and metabolites can influence drug resistance and tumor growth through various mechanisms, primarily by directly affecting their expression and activity or by modulating signaling pathways. Notably, drug-resistant breast cancer often exhibits significantly elevated lactate levels^7^. Glycolytic intermediates can fuel the pentose phosphate pathway, generating ribose-5-phosphate and nicotinamide adenine dinucleotide phosphate (NADPH), which are essential for lipid and nucleic acid biosynthesis. Glutathione (GSH) is a crucial non-enzymatic antioxidant. By maintaining redox balance and mitigating the side effects of chemotherapeutic agents, the production of NADPH enables cancer cells to sustain adequate levels of reduced GSH, which is critical for protecting cells from oxidative damage induced by antitumor therapies^34^,^35^. Pitroda et al. demonstrated that targeting energy metabolic pathways, including glycolysis, can influence tumor cell sensitivity to therapy^36^. Furthermore, these metabolic alterations can promote autophagy in tumor cells while simultaneously inhibiting apoptosis and facilitating EMT. Autophagy, a cellular self-digestion process that enables nutrient recycling during periods of nutrient deprivation or stress, such as that induced by anticancer agents, can enhance the therapeutic resistance of tumor cells. Notably, autophagy-related proteins have been shown to regulate the expression and activity of key glycolytic enzymes, thereby influencing glycolytic metabolism within cancer cells^37^. Conversely, when autophagy is inhibited, tumor cells can upregulate glycolysis to maintain their viability^38^. One of the most important processes by which breast cancer cells develop invasive and metastatic characteristics is EMT. Emerging evidence suggests a strong interplay between glycolysis and EMT, whereby glycolysis can induce EMT, further promoting tumor invasion, metastasis, and drug resistance^11^. Overall, glycolysis plays a pivotal role in the development of drug resistance in breast cancer. A comprehensive understanding of the glycolytic pathway's contribution to cancer cell resistance to anticancer drugs, coupled with targeted therapies aimed at modulating glycolysis, holds significant promise for developing innovative strategies to combat drug resistance (Figure 1, Figure 2).

## The role of glycolysis in endocrine therapy

Glycolysis plays a crucial role in several processes that contribute to endocrine therapy resistance, including energy production, metabolic adaptation of tumor cells, biosynthetic support, and modulation of the tumor microenvironment. As a result, tumor cells undergo glycolytic reprogramming, which complicates breast cancer treatment. Understanding these consequences can facilitate the development of innovative treatment approaches to overcome endocrine medication resistance and improve the therapeutic efficacy of breast cancer treatment.

### Glycolytic genes associated with tamoxifen resistance

Tamoxifen (TAM) is a selective estrogen receptor modulator primarily used to treat estrogen receptor-positive breast cancer. Despite its efficacy, approximately 20-30% of breast cancer patients develop resistance to TAM therapy^39^. The complex mechanisms underlying TAM resistance are not fully understood. Based on current research, TAM resistance is broadly categorized into primary and acquired resistance^40^. Primary resistance is often associated with a lack of ERα expression, while acquired resistance can arise from various mechanisms, including increased activity or levels of ERα co-activators (AIB1), growth factor receptors (EGFR, HERB2, and IGF1R), kinases (AKT and ERK1/2), or linker proteins (BCAR1, c-SRC, and PAK1) following prolonged TAM exposure^11^. Studies have demonstrated that TAM-resistant MCF-7 cells (TAMR-MCF-7) rely more heavily on glycolysis than oxidative phosphorylation for cell proliferation and survival^41^. As a crucial metabolic process, glycolysis not only provides energy for tumor cells but also contributes to the development of TAM resistance.

It has been established that some molecules play a crucial role in the glycolytic process within the TAM resistance mechanism. Fructose-2,6-bisphosphatase 3 (PFKFB3) regulates the intracellular levels of fructose-2,6-bisphosphate, thereby controlling the enzymatic activity of PFK-1^42^. Wang et al. observed that as breast cancer progresses, the total expression levels of PFK-1 increase, accompanied by elevated lactate production and LDH activity, suggesting enhanced glycolytic efficiency in breast tissue. Phosphorylation of PFKFB3 at Ser478 in breast cancer cells stimulates glycolysis and cell proliferation. Additionally, PFKFB3 has been linked to the expression of vascular endothelial growth factor (VEGF-A), a key factor in angiogenesis and distant metastasis, which promotes tumor growth and drug resistance^43^. Furthermore, PFKFB3 can regulate the cell cycle progression of breast cancer cells by downregulating p27 through AKT phosphorylation, which affects ERα stability and modulates sensitivity to endocrine therapy^42^. Kotoowski et al. proposed a novel therapeutic strategy targeting the PELP1/SRC-3 complex, which upregulates both PFKFB3 and PFKFB4. By simultaneously targeting this complex and its associated metabolic pathways, it may be possible to inhibit both breast stem cells and circulating tumor cells in ER^+^ breast cancer^44^, offering a promising approach to overcome chemotherapy and endocrine therapy resistance. Similarly, estrogen levels can directly or indirectly influence IGFBP5 expression, which, in turn, affects the sensitivity of breast cancer cells to TAM^45^. Notably, TAMR-MCF-7 cells overexpress IGFBP5, and knockdown of this protein has been shown to induce TAM resistance^46^.IGFBP5 and PFKFB3 overexpression promotes the production of lactate, pyruvate, and fructose-2,6-bisphosphate (F-2,6-BP) and increases the extracellular acidification rate (ECAR), thereby enhancing glycolytic activity^47^. Additionally, glycogen phosphorylation provides a source of raw materials for glycolysis. The mRNA and protein levels of PYGL, a glycogen phosphorylase isoform, are elevated in TAM-resistant breast cancer cell lines. Reducing PYGL expression in TAMR-MCF-7 cells increases their sensitivity to TAM^48^,^49^. Therefore, the glycolytic pathway plays a critical role in the development of breast cancer drug resistance. A growing body of evidence suggests that glycolysis can induce autophagy, leading to autophagy-related resistance. HK2 has been shown to interact with mTOR and inhibit its activity in TAMR-MCF-7 breast cancer cells^50^. By decreasing mTOR activity, a negative regulator of autophagy, HK2 can enhance autophagic processes, contributing to increased tumor cell resistance to TAM therapy^37^. Beclin-1, a key protein involved in autophagosome formation, interacts with LDHA and activates it to promote autophagy in breast cancer cells, thereby increasing their resistance to TAM^51^.

The metabolic regulation of glycolysis plays a pivotal role in the development of tamoxifen resistance in breast cancer. In TAMR-MCF-7 cells, USP46 is significantly overexpressed, preventing the ubiquitin-mediated degradation of PTBP1^52^. Compared to PKM1, PTBP1 upregulates the expression of PKM2, increasing the PKM2/PKM1 ratio. While PKM1 tends to drive cellular processes towards oxidative phosphorylation, PKM2 functions as a pyruvate kinase isoenzyme^53^,^54^. Consequently, a higher PKM2/PKM1 ratio leads to increased reliance on glycolysis for energy generation. In summary, USP46 stabilizes PTBP1, which enhances glycolysis and subsequently contributes to the development of tamoxifen resistance. Targeting the USP46/PTBP1/PKM2 axis may be a potential strategy to reverse tamoxifen resistance. Studies have also indicated that the G protein-coupled estrogen receptor (GPER) contributes to the promotion of aerobic glycolysis by inducing the transcriptional activity and expression of HIF-1α in TAM-resistant cells. GPER stabilizes HIF-1α by preventing its hydroxylation and subsequent ubiquitin-mediated degradation through the upregulation of C-terminal hydrolase-L1 (UCH-L1) and downregulation of prolyl hydroxylase 2 (PHD2). This mechanism results in the interaction between HIF-1α and UCH-L1 while inhibiting the binding of the HIF-1α/PHD2-pVHL complex^55^,^56^. Furthermore, FOXO3A has been shown to reduce mitochondrial oxidative phosphorylation and glycolysis, thereby inhibiting tumor cell growth and survival by decreasing cellular energy production in breast cancer cells. Overexpression of FOXO3A has been demonstrated to enhance the antiproliferative effects of tamoxifen and restore cellular sensitivity to it^57^. Chu et al. showed that LINC00926 activates the E3 ubiquitin ligase STUB1 to ubiquitinate PGK1, leading to downregulation of its expression. Under hypoxic conditions, LINC00926 expression is inhibited, while PGK1 expression is stimulated, primarily through FOXO3A. The FOXO3A/LINC00926/PGK1 axis plays a crucial role in regulating the proliferation, migration, and glycolytic metabolism of breast cancer cells^58^. SIRT3 is a multifunctional protein that plays diverse roles in various signaling pathways and biological processes. Notably, SIRT3 exerts tumor-suppressive effects through its regulation of glycolysis in metabolic control. Studies by Zu et al. revealed significantly lower levels of SIRT3 and PGC-1α expression in breast cancer cell lines TAMR-MCF-7 and MDA-MB-231^59^. Overexpression of either SIRT3 or PGC-1α in these breast cancer cells significantly reduced lactate production and glucose consumption while increasing the NAD^+^/NADH ratio and decreasing ATP concentration. This suggests that SIRT3 or PGC-1α may suppress glycolytic metabolism to inhibit breast cancer cell growth^59^,^60^. Moreover, under pharmacological stress conditions, SIRT3 has been shown to confer drug resistance through enhancing cellular antioxidant defenses and anti-apoptotic mechanisms. SIRT3 has been demonstrated to mitigate tamoxifen-induced cytotoxicity through deacetylation of antioxidant enzymes(SOD2), thereby enhancing cellular antioxidant defenses and attenuating oxidative stress. Furthermore, SIRT3 contributes to the development of drug resistance by modulating mitochondrial function and regulating key apoptosis-related proteins, ultimately suppressing programmed cell death. SIRT3 has been observed to be abnormally elevated in TAM-resistant breast cancer cells, contributing to tamoxifen resistance by modulating estrogen receptor Erβ activity^61^. Consequently, the PGC-1α/SIRT3 pathway appears to influence tamoxifen resistance through its regulation of glycolytic metabolism and may represent a novel target for overcoming such resistance.

**Figure 3:**
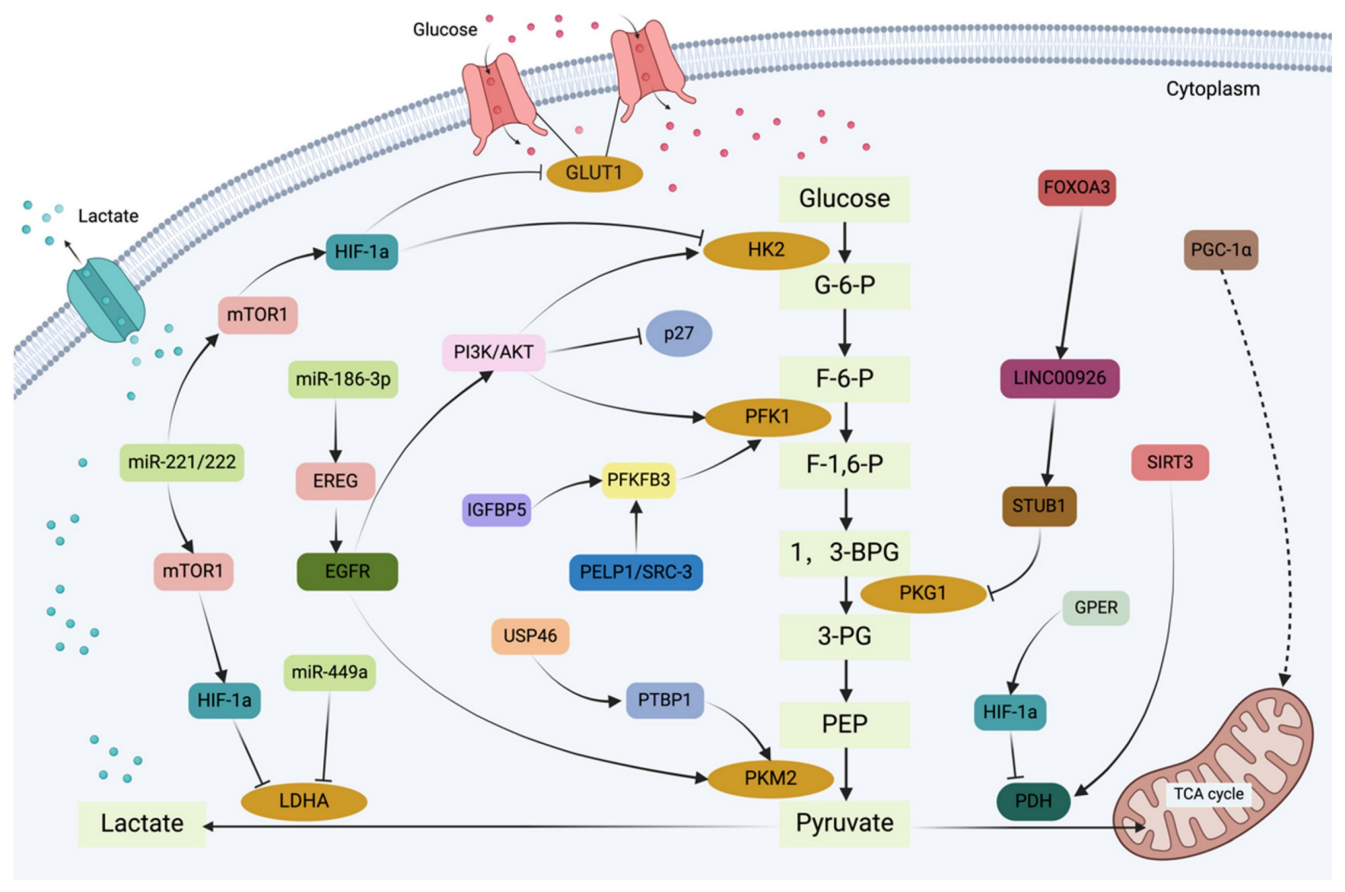
The graphical representation illustrates the glycolysis regulation network in tamoxifen-resistant breast cancer cells. Glycolysis serves as a critical metabolic mechanism in tamoxifen resistance, not only by supplying the energy demands of tumor cells but also by contributing to the development of drug resistance. In breast cancer cells, the glycolysis process is regulated by a number of signaling pathways and transcription factors, ultimately resulting in tamoxifen resistance.

Current evidence suggests that miRNAs mediate the regulation of cellular target genes in the occurrence and development of breast cancer, and a potential correlation may exist between them and the therapeutic resistance of breast cancer. It has been reported that miR-221/222 plays a major role in breast cancer cell resistance to tamoxifen, affecting their susceptibility through various pathways involving key molecules^62^. Besides, miR-221/222 promotes cell proliferation rates and increases the resistance of breast cancer cells to tamoxifen by downregulating p27Kip1 expression, which facilitates the cell cycle progression from the G1 to the S phase. Through the PI3K/AKT, mTORC1, and HIF1α signaling pathways, the overexpression of miR-221/222 significantly lowers the levels of HK2 and LDHA, thereby limiting glycolytic activity. Anti-miR-222/221 treatment of TAMR-MCF-7 cells has been shown to increase their sensitivity to TAM^62^,^63^. Similarly, downregulation of miRNA-449a restores TAM sensitivity in drug-resistant breast cancer cells. Analysis of human lung cancer samples has revealed a strong inverse correlation between LDHA levels and miR-449a expression, suggesting that miR-449a suppresses LDHA production to reduce glycolytic rates^64^,^65^. Finally, the increased glycolysis of TAM-resistant and ER^+^ breast cancer has been associated with the miR-186-3p/EREG axis^66^. Thus, it can be concluded that miRNAs may serve as crucial links between tumor drug resistance and metabolic control, providing novel insights into the management of TAM-resistant breast cancer (Figure 3).

### Glycolytic genes associated with aromatase inhibitor resistance

Aromatase inhibitors (AIs) are a crucial type of endocrine therapy primarily used for postmenopausal individuals with ER^+^ breast cancer. Three primary categories can be used to describe the mechanisms underlying resistance to aromatase inhibitors: genetic factors (such as ESR1 point mutations and amplifications), epigenetic mechanisms, and the interplay of various signaling pathways^67^. The Warburg effect leads to the accumulation of lactate in breast cancer cells. Elevated lactate levels inhibit HDAC activity, resulting in increased histone acetylation. This process promotes glycolytic activity by upregulating the expression of glycolysis-related genes. Notably, HDAC inhibitors have been shown to restore cellular sensitivity to AIs by increasing the expression levels of both ERα and aromatase^68^. Therefore, HDAC inhibitors emerge as a promising therapeutic option for individuals with breast cancer who are resistant to aromatase inhibitors due to the interplay between HDAC activity and glycolysis within tumor cells.

The mechanisms behind resistance to AIs are largely influenced by key enzymes in the glycolytic system. AI-resistant breast cancer cells have been shown to exhibit increased aerobic glycolysis, characterized by elevated PKM2 expression^69^. Silencing PKM2 expression can reduce the glycolytic activity of AI-resistant cells, enhancing the therapeutic efficacy of AIs^70^. In addition to its role in glycolysis, PKM2 is involved in various non-glycolytic processes. As a kinase, it phosphorylates histone H3, promoting carcinogenesis^71^. As a nuclear protein, PKM2 can activate β-catenin^72^. Increased β-catenin expression activates the Wnt/β-catenin signaling pathway, which significantly impacts autophagy and apoptotic processes in breast cancer cells, leading to treatment resistance^33^. Additionally, HK2, a key enzyme in the glycolytic pathway, plays a crucial role in AI-resistant breast cancer. Bacci et al. demonstrated that inhibiting HK2, an enzyme essential for initiating glycolysis, in combination with AIs like letrozole, can synergistically reduce cell viability^73^. Furthermore, AI-resistant breast cancer cells maintain a relatively high NAD^+^/NADH ratio due to enhanced lactate synthesis caused by increased LDHA expression. This equilibrium is essential for maintaining intracellular redox homeostasis and protecting cells from oxidative stress damage. Conversely, oxidative stress induced by suppressing LDHA expression or activity weakens tumor cell resistance to AIs and increases their susceptibility to these drugs^65^,^74^.

In recent years, the mechanism of action of lncRNA DIO3OS in AI-resistant ER^+^ breast cancer has become a focus of research. Chen et al. found that DIO3OS is associated with poor outcomes and is upregulated in AI-resistant breast cancers^17^. Long-term estrogen deprivation leads to increased DIO3OS expression in ER^+^ breast tumor cells of patients undergoing AI therapy. By interacting with polypyrimidine tract-binding protein 1 (PTBP1), DIO3OS stabilizes the mRNA of LDHA through the protection of its 3'UTR. This, in turn, upregulates LDHA expression and initiates glycolytic metabolism in AI-resistant breast cancer cells. By activating the DIO3OS/PTBP1/LDHA cascade, tumor cells may gain metabolic flexibility, enabling them to survive AI therapy^17^,^75^. Consequently, targeting DIO3OS to restore the sensitivity of breast cancer cells to AI treatment may represent a novel therapeutic approach to overcome AI resistance in ER^+^ breast cancer patients.

The most important glucose transporter in breast cancer responsible for glucose uptake is GLUT1^76^. GLUT1's role in promoting glucose uptake is crucial for the malignant transformation and progression of breast cancer^77^. For instance, microRNA-140-5p suppresses breast cancer glycolysis by targeting GLUT1^78^. The PI3K/AKT/mTOR signaling pathway is upregulated in breast cancer, contributing to both aerobic glycolysis and treatment resistance. Abdel et al. demonstrated that this pathway enhances glycolytic activity by inducing the expression and plasma membrane translocation of GLUT1^31^. Mutations in genes such as PIK3CA, S6K1, 4E-BP1, and PTEN can lead to persistent activation of the PI3K/AKT/mTOR signaling pathway^79^-^81^. By enabling tumor cells to bypass estrogen-dependent pathways, these gene mutations promote tumor growth, cell proliferation, and endocrine resistance in breast cancer. Notably, PIK3CA mutations are frequently observed in HER2^+^ and ER^+^ breast cancer subtypes. A preclinical study showed that PI3K inhibitors can prevent the development of acquired endocrine resistance and induce apoptosis in ER^+^ breast cancer cells with PIK3CA mutations under estradiol deprivation^82^. Combining fulvestrant with PI3K inhibitors has been shown to reverse AI resistance in previously resistant cells and induce tumor cell death. Therefore, combination therapy of PI3K inhibitors and fulvestrant offers a promising therapeutic approach for AI-resistant breast tumors with PIK3CA mutations.

Compared to normal breast tissues, breast cancer tissues exhibit significantly higher levels of miR-155 expression^73^. By altering the expression of apoptosis-related proteins, miR-155 influences the sensitivity of breast cancer cells to AIs. Specifically, miR-155 can enhance the anti-apoptotic effects observed in drug-resistant cells by upregulating the expression of Bax and activated Caspase-3 while downregulating the apoptosis-inhibitory gene BCL-2. Additionally, miR-155 stimulates the production of drug-resistance proteins associated with breast cancer, including Multidrug Resistance Protein 1 (MRP1), P-glycoprotein (P-gp), and Breast Cancer Resistance Protein (BCRP)^83^,^84^. This process promotes cellular drug efflux, exacerbating acquired drug resistance. Beyond its role in regulating apoptosis, miR-155 plays a crucial role in glycolysis. It facilitates metabolic reprogramming essential for tumor growth in breast cancer cells by modulating the balance between glycolysis and oxidative phosphorylation (OXPHOS). miR-155 upregulates HK2 and monocarboxylate transporter 4 (MCT4), thereby increasing glycolytic activity and promoting rapid tumor growth^85^. Given that negative outcomes during AI therapy are associated with high baseline levels of miR-155, it can be concluded that miR-155 may serve as a therapeutic target for AI resistance as well as a diagnostic biomarker. Assessing its efficacy and safety, along with the development of specific inhibitors targeting miR-155, may offer novel approaches to combat AI resistance.

The aforementioned findings clearly indicate that these genes and enzymes are crucial for glycolytic pathways and the pathophysiology of breast cancer. Understanding their roles will facilitate the development of novel treatment strategies targeting aromatase inhibitor-resistant breast cancer patients.

### Glycolysis-mediated drug resistance in hormone receptor-positive breast cancer

CDK4/6 inhibitors are essential for preventing cancer cell growth by blocking the activity of cyclin-dependent kinases 4 and 6, particularly in the treatment of advanced ER^+^ and HER2-negative breast cancer^86^. The molecular mechanisms of resistance to CDK4/6 inhibitors primarily involve three aspects, one of which is the activation of bypass pathways closely linked to glycolysis. By activating the mTOR signaling pathway, cancer cells can continue to support cell survival and proliferation, circumventing the inhibitory effects of CDK4/6 inhibitors. When tumor cells undergo glycolysis, signaling pathways such as PI3K/AKT, RAS/RAF, MEK/ERK, and others are also activated^87^,^88^. As a result, the glycolytic pathway plays a crucial role in the mechanism of CDK4/6 resistance.

Glycolysis-related glucose transporters and key enzymes are activated in breast cancer cells resistant to CDK4/6 inhibitors, promoting the glycolytic process. This activation fuels cancer cell growth, proliferation, and energy production. mTOR, comprising two complexes, mTORC1 and mTORC2, functions as a downstream effector of AKT. AKT phosphorylates multiple sites on TSC2, inhibiting its GAP function and leading to increased Rheb-GTP levels, which in turn activates mTORC1^89^,^90^. mTORC1 upregulates HIF-1α expression and facilitates the metabolic shift from OXPHOS to glycolysis^91^. mTORC1 can directly regulate key glycolysis-related enzymes and glucose transporters. mTORC2 promotes glycolysis and glucose uptake through the activation of AKT and GLUT1. Overactivation of the mTOR pathway promotes cell division and growth, which is directly linked to tumor development and progression. Through downstream effectors like S6K1 and 4E-BP1, the mTOR signaling pathway either stimulates the translation of cell cycle regulatory proteins such as Cyclin D and Cyclin E or induces the expression of P-gp. This activation leads to aberrant cell proliferation and chemotherapy-resistant cancer cells^40^,^79^,^92^. Furthermore, targeting mTOR kinase can impact both CDK4/6 inhibitor resistance pathways and glycolytic processes. Inhibiting mTOR kinase through targeted therapy represents a novel therapeutic approach to overcome CDK4/6 inhibitor resistance. In ribociclib-resistant breast cancer cells, CDK4/6 inhibitors activate the PI3K/AKT pathway and phosphorylate AKT via PDPK1. PI3K overexpression increases Cyclin D1 expression, further contributing to CDK4/6 inhibitor resistance^93^. Combining CDK4/6 inhibitors with PI3K/mTOR inhibitors can significantly slow tumor growth in animal models, suggesting that PI3K/mTOR inhibitors can help restore sensitivity to CDK4/6 inhibitors.

One mechanism by which breast cancer cells develop resistance to CDK4/6 inhibitors involves the degradation or mutation of the retinoblastoma (RB) protein. The RB protein inhibits cell cycle progression from the G1 to S phase by suppressing the activity of E2F transcription factors. Activation of E2F transcription factors leads to overexpression of HK2, PFK1, and LDHA, thereby increasing glycolytic activity^86^. Loss of RB protein function results in excessive activation of E2F transcription factors and the Cyclin E-CDK2 complex, leading to compensatory activation of the PI3K/AKT/mTOR signaling pathway. This bypasses the effects of CDK4/6 inhibitors and allows for continued cell proliferation. This pathway is involved in both aerobic glycolysis and breast cancer development. Therefore, combining CDK4/6 inhibitors with targeted inhibitors targeting the RB protein may enhance therapeutic efficacy. HIF-1α promotes glycolysis by upregulating HK2 and GLUT1. The oncogenic activities of elevated AKT and mTOR also promote HIF-1α expression, leading to the continuous transcription of glycolysis-driving enzymes and lactate production^94^. The RB protein can downregulate the expression of glycolysis-related genes by inhibiting HIF-1α stability or function. Reduced RB protein function results in increased HIF-1α activity, leading to aberrant stimulation of the glycolytic pathway^95^. These findings suggest that tumor cells with defective RB protein activity utilize the glycolytic pathway as an alternative energy source to withstand drug-induced stress, ultimately leading to resistance to CDK4/6 inhibitors. Therefore, targeting the RB protein, such as through SETDB1 inhibitors, to prevent its degradation or gene alterations may enhance the efficacy of CDK4/6 inhibitors by interfering with the glycolytic process and overcoming drug resistance.

## The role of glycolysis in HER2-positive targeted therapy

15-20% of breast cancers exhibit HER2 overexpression, making it a notorious tyrosine kinase receptor^96^. HER2 activation can lead to the activation of the PI3K-Akt-mTOR signaling pathway, upregulating enzymes and transporters associated with the glycolytic pathway. This facilitates energy generation through glycolysis under hypoxic conditions, supporting rapid tumor cell proliferation and growth^97^,^98^. Consequently, targeting both HER2 signaling and the glycolytic pathway with HER2-targeted medications and glycolytic inhibitors can more effectively prevent tumor development and progression. We next discuss the mechanisms of glycolysis in several HER2-targeted medications that positively regulate breast cancer resistance.

### Glycolysis-related mechanisms in trastuzumab resistance

Trastuzumab is a targeted treatment medication for HER2^+^ breast cancer. Targeted therapy resistance can arise from alterations in the HER2 protein's structure, such as *HER2* gene mutations and nuclear localization. Additionally, p27kip1 downregulation, PI3K signaling pathway activation, PTEN loss, and signal transduction through alternative receptors can contribute to resistance^99^. Downstream signaling pathways, including the PI3K-Akt-mTOR pathway, the Ras-Raf-MEK-ERK pathway, and the IGF-IR pathway, can become abnormally and persistently activated, enabling tumor cells to evade trastuzumab's effects and develop drug resistance^100^,^101^.

*PTEN*, a tumor suppressor gene, possesses dual phosphatase activity, allowing it to preferentially dephosphorylates PIP3 to PIP2, thereby inhibiting the activation of downstream effector molecules like PDK1 and AKT by PIP3^80^. PTEN normally suppresses AKT activation by reducing signaling from G protein-coupled receptors (GPCRs) and receptor tyrosine kinases (RTKs). This prevents downstream signaling events regulated by AKT and negatively regulates the PI3K/Akt pathway by dephosphorylating PIP3. In contrast, mutations or deletions in the *PTEN* gene can lead to hyperactivation of the PI3K-Akt-mTOR pathway. This aberrant activation upregulates transcription factors and associated glycolysis-related enzymes^102^. Additionally, the *PTEN* gene has been shown to function as a protein phosphatase, interacting with PGK1, dephosphorylating it, and reducing its activity, thereby effectively inhibiting the glycolytic process and ATP production. Zhao et al. demonstrated that in ErbB2^+^ cancer cells, trastuzumab can downregulate LDHA and heat shock factor 1 (HSF1), inhibiting glycolysis and suppressing tumor growth. Trastuzumab resistance can develop due to increased glycolysis resulting from elevated HSF1 and LDHA levels^103^. Similarly, several glycolytic genes contribute to the development of trastuzumab resistance. Specifically, Ferla et al. identified the *ANKRD44* gene as a potential contributor to trastuzumab resistance^104^. This gene encodes a protein containing ankyrin repeat sequences, which are involved in crucial biological processes such as intracellular signal transduction, cell cycle regulation, and gene expression modulation, suggesting that ANKRD44 may facilitate the emergence of resistance by altering the intracellular signaling network. Furthermore, Yuan et al. identified *NDUFA4L2* as a gene that promotes trastuzumab resistance in HER2-positive breast cancer^105^. High expression of NDUFA4L2 in HER2-positive breast cancer cells may enable cells to maintain energy supply in hypoxic environments and simultaneously reduce ROS levels.

The overexpression of t-DARPP may mediate trastuzumab resistance through AKT activation^106^. t-DARPP has been found to activate IGF-1R signaling by heterodimerizing with EGFR and HER2, upregulating the capacity to regulate glycolysis in HER2^+^ breast cancer^100^. Concurrently, Liu et al. demonstrated that ALKBH5-mediated m6A demethylation of GLUT4 mRNA enhances glycolytic activity in breast cancer cells and contributes to resistance against HER2-targeted therapies^107^. Furthermore, Wang et al. discovered that disruption of the circadian rhythm via the PER1-HK2 axis could reverse trastuzumab resistance in gastric cancer. PER1 protein expression levels in breast cancer are strongly associated with ER, PR, c-erbB2, and histological grade^98^. Therefore, it is plausible that diurnal oscillations in glycolytic activity also occur in trastuzumab-resistant HER2^+^ breast cancer. Targeting this glycolytic cycle may represent a novel strategy to help patients with trastuzumab resistance.

### Mechanisms associated with glycolysis in TKI resistance

Tyrosine kinase inhibitors (TKIs) are widely used to treat individuals with HER2^+^ breast cancer. However, prolonged exposure to TKIs can lead to tumor cell resistance. A major cause of this resistance is metabolic dysregulation within tumor cells, impacting cell growth, senescence, apoptosis, and proliferation at various levels of gene expression, particularly through alterations in glycolytic pathways. TKIs have been shown to specifically downregulate HK2, PKM2, and GLUT1, three key enzymes and transporters involved in glycolysis^108^.

Breast cancer cells can develop resistance to lapatinib through several mechanisms, often linked to *PIK3CA* mutations and *PTEN* deletions. Approximately 30% of patients with HER2^+^ breast cancer harbor activating PIK3CA mutations. These alterations trigger the PI3K/AKT/mTOR signaling pathway, increasing glycolysis and providing the energy necessary for tumor cell survival and growth^82^. Recent research has identified a novel proteolytic targeted chimera (PROTAC) that selectively targets PI3K-p110α. By degrading this protein, it has been shown to inhibit breast cancer cell growth. Moreover, even at very low concentrations, it can resensitize lapatinib-resistant cell lines. Compared to conventional PI3K inhibitors, the PROTAC molecule exhibits stronger anti-tumor effects and significant anti-proliferative activity in two lapatinib-resistant breast cancer cell lines, MDA-MB-453 and JIMT1^79^,^82^. Additionally, PTEN loss promotes PI3K signaling and, through the BMX/STAT3 pathway, contributes to tumor cell survival and metastasis. Research suggests that PI3Kβ, rather than PI3Kα, is a key driver of STAT3 activation in the absence of PTEN, facilitating tumor immune evasion and ultimately leading to drug resistance^102^. Furthermore, glycolysis can enhance tumor resistance to targeted therapy by promoting autophagy. Studies have shown that autophagy can increase the resistance of HER2^+^ breast cancer to lapatinib therapy^109^. EGFR activation can accelerate glycolysis and upregulate the expression of essential enzymes. By preventing autophagy-mediated EGFR degradation, increased glycolysis can sustain high EGFR expression and further support tumor cell survival and proliferation. Research indicates that EGFR-TKI treatment can induce autophagy in various tumor cells, potentially leading to tumor resistance^110^.

### The Correlative Mechanisms of Glycolysis in Resistance to ADCs

**Figure 4:**
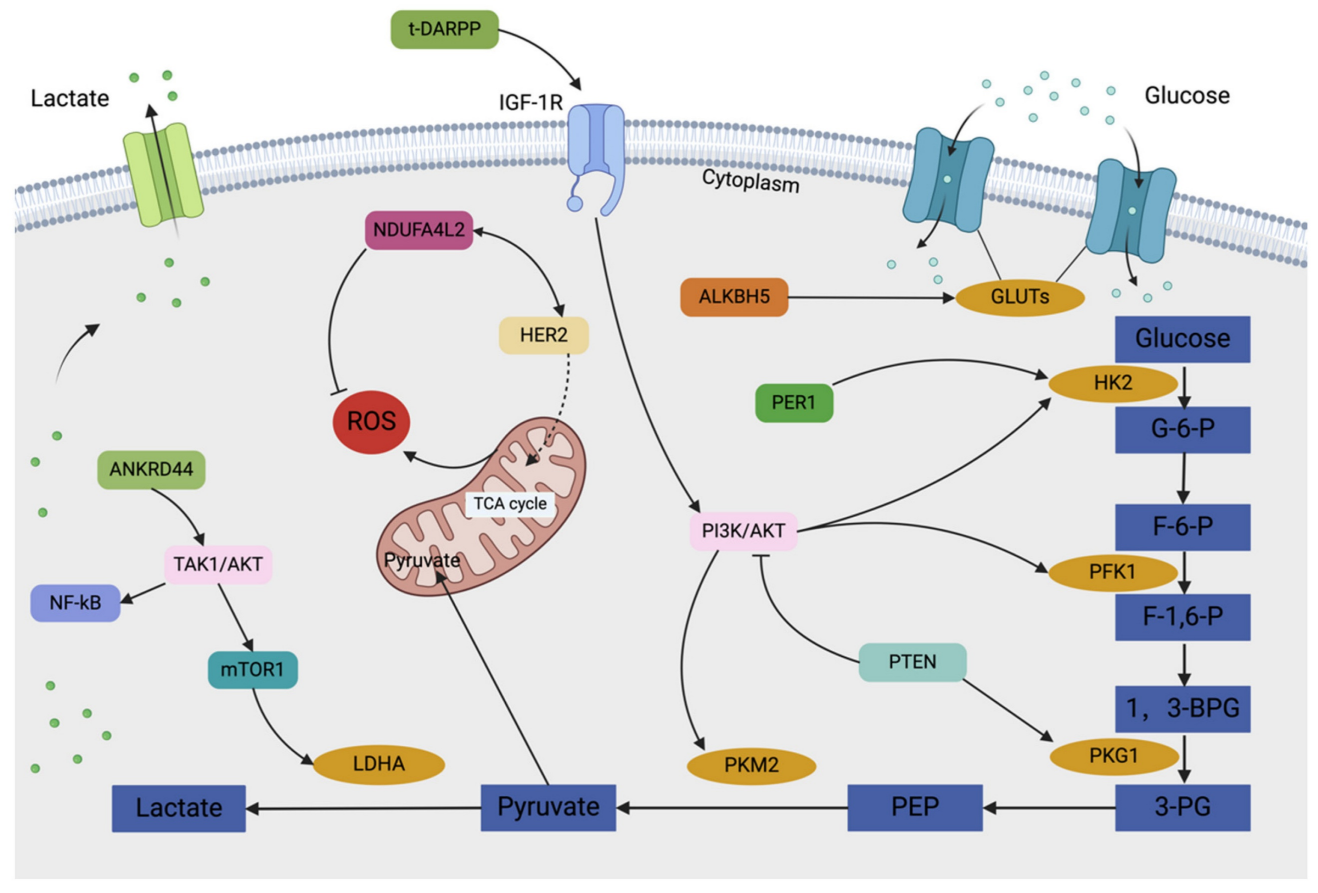
The graphical illustration presents the glycolysis regulatory network in drug resistance of HER2-positive breast cancer. Under hypoxic conditions, HER2-positive breast cancer cells rely on the glycolytic pathway for energy production, thereby supporting their rapid proliferation and growth. Certain signaling pathways and regulators can promote glycolysis and enhance energy metabolism, thereby leading to cellular drug resistance.

Antibody-Drug Conjugates (ADCs) comprise cytotoxic drugs, linkers, and monoclonal antibodies. They leverage the targeting capabilities of antibodies to deliver therapeutic agents directly to tumor cells. Upon internalization, cytotoxic drugs are released intracellularly to exert their lethal effects. While this approach holds promise as a potential anti-tumor strategy, drug resistance remains a significant challenge. Multiple complex mechanisms contribute to ADC resistance. Recent studies have identified several key factors, including alterations in antigen expression, aberrant activation of downstream signaling pathways, decreased lysosomal protein proteolytic activity, and increased drug efflux pump expression^111^. Tumor cells often overexpress important glycolytic enzymes and exhibit aberrant activation of various signaling pathways, promoting glycolysis and generating substantial energy for tumor cell growth. In addition to upregulating the expression and activity of drug efflux pumps (such as P-gp and multidrug resistance-associated protein), glycolytic byproducts can also induce drug efflux from cells, reducing intracellular drug accumulation. This phenomenon exacerbates drug resistance, hindering the achievement of therapeutic efficacy^112^. Furthermore, lactic acid accumulation not only alters the tumor microenvironment, making it more conducive to immune evasion but also affects intracellular signal transduction pathways that support tumor cell growth and drug resistance. Lactic acid accumulation alters intracellular pH levels, impacting lysosomal acidification and enzymatic activity. This change affects the internalization and degradation processes associated with ADCs, preventing the release of cytotoxic drugs and ultimately leading to increased drug resistance. Therefore, targeting the glycolytic process is a critical component of overcoming ADC resistance. Combining ADC therapies with targeted glycolysis inhibitors can more comprehensively disrupt tumor cell metabolism, overcoming the limitations of individual treatments and improving therapeutic outcomes. Research has shown that T-DM1 and 2-DG synergistically inhibit the growth and proliferation of HER2-positive breast cancer cells^111^. Furthermore, by inhibiting the activation of the Ras/Raf/MEK/ERK/MAPK and PI3K/AKT/mTOR pathways, lactate production, and tumor cell survival can be reduced, enhancing the therapeutic efficacy of ADCs (Figure 4).

## Therapeutic potential of glycolysis in breast cancer

**Figure 5:**
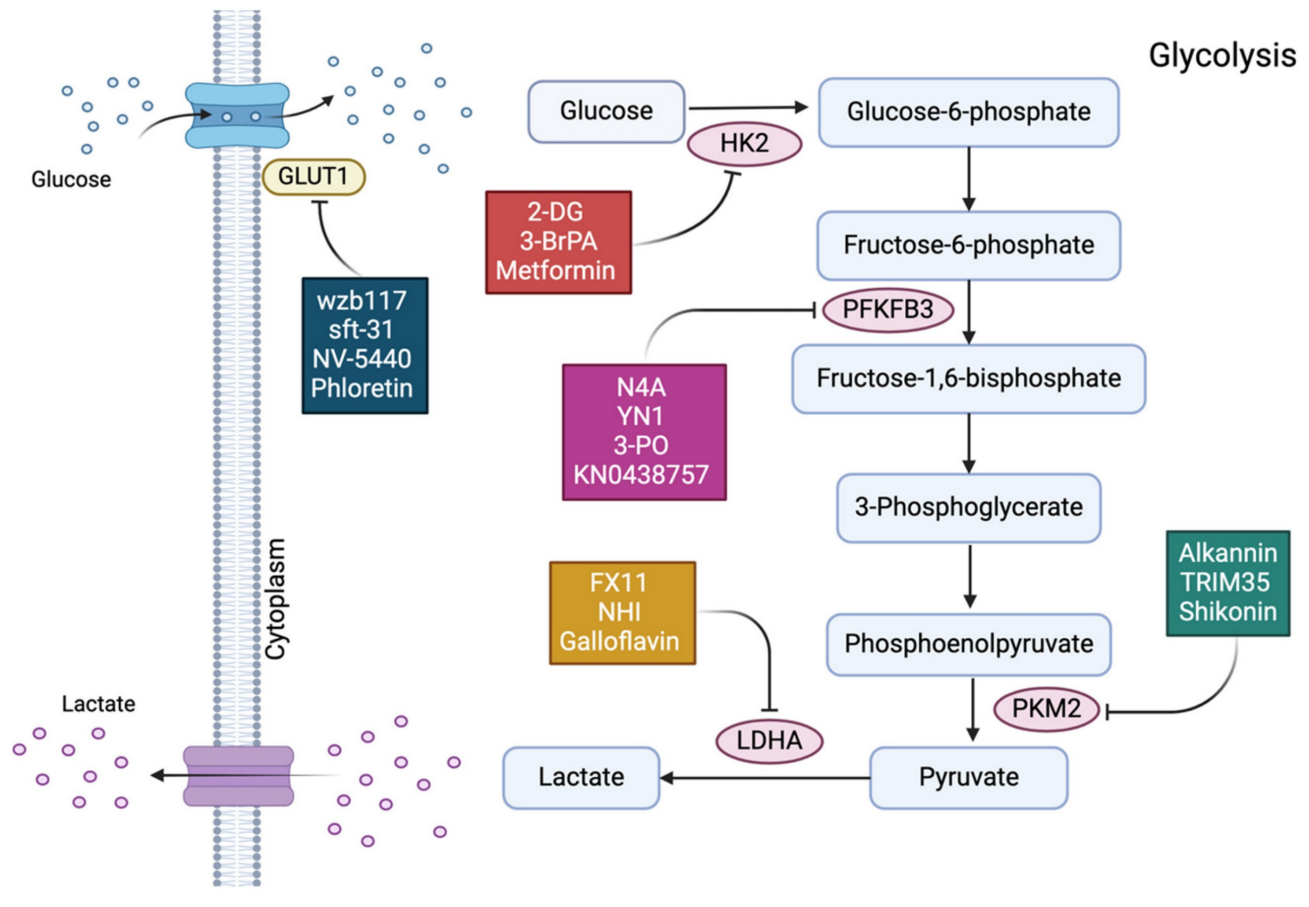
In different subtypes of breast cancer (excluding triple-negative), potential inhibitors of different glycolytic enzymes are described in boxes. Targeting the glycolytic pathway can improve the therapeutic efficacy and reverse the current state of treatment resistance for breast cancer. Inhibitors of glycolytic enzymes GLUT-1, HK2, PFKFB-3, PKM2, and LDHA can affect the energy metabolism of cancer cells by impeding the glycolysis process.

Given its diverse molecular mechanisms in breast cancer resistance, targeting glycolysis may offer a novel therapeutic perspective for breast cancer. As previously established, targeting glucose transporters and key glycolysis-related enzymes and regulators has been shown to effectively inhibit the glycolytic pathway, thereby preventing treatment resistance in various breast cancer subtypes. For example, HK, the first rate-limiting enzyme in the glycolytic pathway, binds to the outer mitochondrial membrane and catalyzes the phosphorylation of glucose to produce glucose-6-phosphate (G-6-P). By selectively inhibiting HK activity, glycolysis in cancer cells can be impeded, effectively reducing G-6-P synthesis. Three currently available HK inhibitors, 2-DG, Metformin, and 3-BrPA, have demonstrated remarkable efficacy in restoring sensitivity in drug-resistant breast cancer cells^113^-^116^. The glycolytic process relies on the enzyme PFKFB-3, which has been the subject of numerous synthetic inhibitor studies. YN1, 3-PO, KN0438757, and N4A are known synthetic inhibitors of PFKFB-3^117^-^119^. The final rate-limiting step of glycolysis is catalyzed by PKM2, another important therapeutic target within the glycolytic pathway. Alkannin, TRIM35, and Shikonin are PKM2 inhibitors^120^-^122^. Pyruvate is converted to lactate by LDH, which has emerged as a promising target for tumor therapy. Numerous studies have focused on inhibitors targeting LDHA, with notable examples including FX11, galloflavin, and NHI^123^-^125^. Additionally, as transport proteins in the glycolytic pathway, GLUTs are essential, with GLUT1 being particularly important in breast cancer cells. Key inhibitors targeting GLUT1 include phloretin, NV-5440, sft-31, and WZB117^126^-^130^. Furthermore, HIF-1α directly inhibits oxidative phosphorylation and the tricarboxylic acid cycle. The HER-2/neu signaling pathway has been shown to upregulate HIF-1α, which in turn promotes cellular glycolysis. Consequently, HIF-1α inhibitors may effectively reduce glycolysis and represent a viable treatment approach for overcoming breast cancer resistance^131^-^133^. Finally, 1,25(OH)2D3 can inhibit the activity of PFK-1, thereby slowing down the glycolytic process, resulting in a decrease in intracellular ATP levels and subsequently promoting cell apoptosis. J.M. Santos et al. noted in their study that vitamin D3 is essential for regulating glucose metabolism and glycolytic enzymes in breast cancer cells. By altering actin expression and promoting cell stiffness, vitamin D3 therapy can reverse the EMT phenotype of breast cancer^134^. Apart from the inhibitors of the aforementioned relevant enzymes and transcription factors, numerous targets within the glycolytic pathway play decisive roles. However, research on inhibitors targeting these specific sites is currently limited. With a growing understanding of the functions, mechanisms, and regulation of glycolysis in breast cancer resistance, we anticipate the development of more and more glycolysis-targeting inhibitors in the near future to transform the current landscape of breast cancer resistance treatment (Figure 5, Table 1).

## Conclusion and perspectives

Breast cancer, a prevalent malignant neoplasm posing a significant threat to women's health, presents numerous challenges in treatment. The mechanisms underlying the development of endocrine and targeted therapy resistance in breast cancer cells are intricate and involve the interplay of various factors, though the specific mechanisms remain incompletely elucidated. Glycolysis, a vital pathway for breast cancer cells to acquire energy, plays a crucial role in promoting tumor growth, invasion, metastasis, and the development of therapeutic resistance. The glycolytic process in breast cancer cells is closely associated with endocrine and targeted therapy resistance. This article provides a systematic review of the role of glycolysis in the metabolic reprogramming of breast cancer, along with the mechanisms by which enzymes and transcription factors within the glycolytic pathway contribute to the development of resistance to endocrine therapy and HER2^+^ targeted therapy. A deeper understanding of glycolytic genes and their regulatory mechanisms will not only shed light on the molecular basis of glycolysis in the development of breast cancer resistance but also provide a theoretical foundation for the development of novel therapeutic strategies. With a growing body of research on glycolytic inhibitors, the combination of these inhibitors with existing anticancer drugs is anticipated to significantly improve treatment outcomes for breast cancer patients and reduce the incidence of drug resistance. However, achieving this goal necessitates continuous exploration and validation at the cellular, animal, and clinical levels through conducting relevant basic research to uncover the mechanisms of glycolysis in endocrine and targeted therapy resistance of breast cancer. Furthermore, clinical trials are essential to verify the feasibility of targeting glycolytic genes for the treatment of breast cancer resistance. By detecting resistance markers during treatment and utilizing these new markers, early diagnosis of the resistant population and early identification of high-risk populations for resistance can benefit a greater number of breast cancer patients. Future research may involve combining targeted glycolytic inhibitors with existing breast cancer drugs, which could help overcome the development of therapeutic resistance and improve outcoms for a larger number of breast cancer patients.

## Figures and Tables

**Table 1:** Antiglycolytic medications that target the transcription factors, transporter proteins, and associated enzymes in glycolysis

Target	Inhibitor	Target nature	Mechanism of Action	Reference
HK2	2-DG	Synthetic glucose analogues	2-DG-6-P, a nonmetabolizable product of 2-DG, accumulated and feedback regulation of HK2.	110
	3-BrPA	Brominated derivative of pyruvate	3-BrPA disrupts the binding of HK2 to mitochondria through voltage-dependent anion channel protein and triggering the intrinsic pathway of apoptosis.	109,108
	Metformin	Synthetic drug	Metformin can reduce the generation of G6P and is an allosteric inhibitor of HK2.	107
PFKFB-3	N4A	Synthetic competitive Inhibitor	N4A forms a hydrogen bond with Arg74, Asp124, Thr126, and Arg132 to attach to the Fru-6-P binding pocket of the PFKFB-3 kinase domain.	113
	YN1	Synthetic small molecule inhibitor	competing with the substrate by occupying the PFKFB-3's Fru-6-P binding site.	113
	3-PO	Small molecule inhibitor	Reduced glycolytic flow results from being bound to the PFKFB-3 substrate binding pocket and competing with Fru-6-P.	112
	KN0438757	Synthetic inhibitor	Prevent PFKFB-3's enzymatic function in DNA repair and homologous recombination, and make the altered cells more sensitive to radiation.	111
PKM2	Alkannin	Natural compound	Effectively bind to PKM2 and inhibit the cellular glycolytic flow.	116
	TRIM35	Synthetic Peptide	Warburg effect suppression results from interacting with PKM2 through the coiled-coil domain and preventing the phosphorylation of the Y105 residue.	114
	Shikonin	Natural small molecules	Hydrogen bonds (Leu352, Lys310, Tyr389, Ala387, and His28) bind to the allosteric region of PKM2.	115
LDHA	Galloflavin	Derivative of gallic acid	Pyruvate and NADH are not in competition with the inhibitor when it binds to the free enzyme. The carbonyl and hydroxyl group of Galloflavin form hydrogen bond with LDHA.	117
	FX11	Small molecule inhibitor (Gossypol analogues)	Competitive with the cofactor of reduced NADH	118
	NHI	Synthetic compound	Competitive with pyruvate and NADH due to its binding pocket.	119
GLUT1	wzb117	Small molecule inhibitor	Reversibly attaching to the exofacial sugar binding sites of GLUT1	123,77
	sft-31	Small molecule inhibitor	Modify the substrate binding residues to inhibit GLUT1 in von Hippel-Lindau deficient renal cancer cells. STF-31 inhibits the function of the glucose transporter by binding to the central pore of GLUT1 and perhaps interacting with two residues, Arg126 and Trp412.	126,128
	NV-5440	Small molecule Inhibitor	The benzonitrile group of NV-5440 interacts hydrophobically with Val165 of the glucose binding sites of GLUT1 and generates hydrogen bonds with Gln282 and Trp388.	124
	Phloretin	Natural Compound	binds to the GLUT1 exofacial vestibule. With three hydrogen bonds, its phenol ring seems to be located in the same pocket as glucose.	121
HIF-1a	PX-478	Synthetic compound	Reduce the amount of HIF-1α mRNA, inhibit translation, and inhibit the protein level and transactivation of HIF-1α.	131
	Melatonin	Indole heterocyclic compound	Promote the degradation of HIF-1α	133
	Curcumin	Natural phenolic compound	Promote the degradation of HIF-1α	133
	YC-1	Small molecule inhibitor	Inhibit the PI-3K/Akt/mTOR/4E-BP pathway	132

## Abbreviations

HER-2: human epidermal growth factor receptor 2
PR: progesterone receptor
ER: estrogen receptor
PGK1: phosphoglycerate kinase 1
EMT: epithelial-mesenchymal transition
PFK-1: phosphofructokinase-1
PKM2: pyruvate kinase 2
lncRNA: long non-coding RNA
LDHA: lactate dehydrogenase A
TAMs: tumor-associated macrophages
EVs: extracellular vesicles
GLUT1: glucose transporter 1
GLUT3: glucose transporter 3
HK: hexokinase
PFK: phosphofructokinase
PKM1/2: pyruvate kinase M1/2
LDH: lactate dehydrogenase
NADH: nicotinamide adenine dinucleotide
HIF-1: hypoxia-inducible factor-1
AMPK: AMP-activated protein kinase
NADPH: nicotinamide adenine dinucleotide phosphate
GSH: glutathione
TAM: tamoxifen
PFKFB3: fructose-2,6-bisphosphatase 3
VEGF-A: vascular endothelial growth factor A
F-2,6-BP: fructose-2,6-bisphosphate
ECAR: extracellular acidification rate
GPER: G protein-coupled estrogen receptor
UCH-L1: upregulation of C-terminal hydrolase-L1
PHD2: prolyl hydroxylase 2
Ais: aromatase inhibitors
PTBP1: polypyrimidine tract-binding protein 1
MRP1: multidrug Resistance Protein 1
P-gp: P-glycoprotein
BCRP: breast cancer resistance protein
OXPHOS: oxidative phosphorylation
MCT4: monocarboxylate transporter 4
RB: retinoblastoma
GPCRs: G protein-coupled receptors
RTKs: receptor tyrosine kinases
HSF1: heat shock factor 1
TKIs: tyrosine kinase inhibitors
PROTAC: proteolytic targeted chimera
ADCs: antibody-drug conjugates
G-6-P: glucose-6-phosphate
